# *Mycoplasma arginini* Cellulitis, Tenosynovitis, and Arthritis in Kidney Transplant Recipient, Slovenia, 2024

**DOI:** 10.3201/eid3106.250149

**Published:** 2025-06

**Authors:** Tjaša Vivoda, Tereza Rojko, Barbara Kokošar Ulčar, Katja Strašek Smrdel, Andraž Celar Šturm, Darja Keše, Tina Triglav, Željka Večerić Haler

**Affiliations:** University Medical Center Ljubljana, Ljubljana, Slovenia (T. Vivoda, T. Rojko, B. Kokošar Ulčar, Ž. Večerić Haler); Faculty of Medicine, University of Ljubljana, Ljubljana (T. Vivoda, T. Rojko, K. Strašek Smrdel, D. Keše, T. Triglav, Ž. Večerić Haler); Institute of Microbiology and Immunology, Ljubljana (K. Strašek Smrdel, A. Celar Šturm, D. Keše, T. Triglav)

**Keywords:** Mycoplasma arginini, bacteria, zoonoses, cellulitis, arthritis, immunocompromised, kidney transplant recipient, Slovenia

## Abstract

*Mycoplasma arginini* is a bacterium primarily found in animals and is seldom reported in human infections. We identified *M. arginini* infection in a severely immunocompromised kidney transplant recipient in Slovenia. Clinicians should be aware of *M. arginini’s* potential as a pathogen in immunocompromised persons with animal contact.

*Mycoplasma* species belong to the class Mollicutes, a group of bacteria characterized by lack of a cell wall. Those organisms are among the smallest organisms capable of autonomous replication and measure ≈0.3–0.8 μm. 

*M. arginini* is a common colonizer in respiratory and urogenital tracts of various animals, including cats, dogs, cattle, and sheep, and is generally considered to be of low pathogenicity (*1*). However, in immunocompromised persons, *M. arginini* infection can lead to severe complications (*2*–*4*; M.A. May et al., unpub. data, https://www.preprints.org/manuscript/201809.0533/v1). We report a case of *M. arginini* infection in a kidney transplant recipient in Slovenia.

## The Study

A 56-year-old woman was seen for a 3-week history of swelling, redness, and pain in her left forearm. She had a 17-year history of IgG kappa plasmacytoma and C3 glomerulonephritis, which led to end-stage renal failure and 2 kidney transplants. She underwent her first kidney transplant in 2011 and then a second transplant in 2022 after failure of the first graft. Both transplants were complicated by C3 glomerulonephritis recurrence. Approximately 6 months after the second kidney transplant, she started treatment with high-dose intravenous methylprednisolone, rituximab (chimeric monoclonal antibody targeting CD20 on B cells), and plasmapheresis, in addition to standard immunosuppressive therapy (tacrolimus, mycophenolate mofetil, and methylprednisolone). During that intensive immunosuppression, opportunistic infections developed, including cutaneous *Alternaria alternata* fungi infection of the left shin and cytomegalovirus reactivation, which necessitated adjustments to her immunosuppressive regimen. Over the next 2 years, declining graft function caused by C3 glomerulonephritis was managed with ravulizumab (a monoclonal antibody targeting complement component 5) and plasmapheresis. Granulocyte colony-stimulating factors were intermittently administered to treat episodes of neutropenia.

Approximately 2 years after her second kidney transplant, she noticed a small lump in the left mid-forearm with redness that progressively spread toward her wrist and elbow. Her doctors prescribed oral amoxicillin/clavulanic acid. After 5 days, that treatment failed to resolve her worsening symptoms, which prompted hospitalization. At admission, she exhibited swelling, redness, and restricted joint mobility in the left wrist. The patient had frequent contact with household cats and a dog and reported having sustained a cat bite at the site of small lump in the mid-forearm a week before admission.

Laboratory findings showed elevated C-reactive protein and β-d-glucan levels, normal procalcitonin level, and normal leukocyte count. Immune status evaluation using QuantiFERON Monitor (QIAGEN, https://www.qiagen.com) showed a moderate cell-mediated immune response, elevated plasma Torque Teno virus DNA (167,000 copies/mL), substantially decreased B-lymphocyte counts (likely caused by rituximab), and severe hypogammaglobulinemia (Table; Appendix). An ultrasound confirmed cellulitis, tenosynovitis of carpal extensor tendons, and arthritis of the radiocarpal joint (Appendix Figure 1). 

**Table:** Laboratory values for *Mycoplasma arginini* cellulitis, tenosynovitis, and arthritis in kidney transplant recipient, Slovenia, 2024*

Variable	Sample collection				Reference range
	At clinic†	At admission	At discharge	After treatment‡	
White blood count, cells × 10^9^/L	5.5	4.2	5.5	6.8	4.0–10.0
C-reactive protein, mg/L	5	40	13	<5	<5
Procalcitonin, µg/L	0.6	0.10	0.05	NA	<0.24
IgG, g/L	NA	2.7	14.7§	6.1	7.67–15.90
IgA, g/L	NA	<0.24	<0.24	NA	0.61–3.56
IgM, g/L	NA	<0.17	0.24	NA	0.37–2.86
β-d-glucan, pg/mL	189.3	207.2	170.2	192	<59.9
GM antigen, ELISA index	0.06	0.12	NA	NA	<0.5
Cryptococcal antigen, ELISA titer	1:0.03	1:0.04	NA	NA	<1:1
Blood cultures¶	NA	Negative	NA	NA	Negative

*IgG values were adjusted on the basis of presence of known IgG/κ spike. GM, galactomannan; NA, not available. †The patient’s clinic at symptom onset. ‡Treatment included a completed 6-week course of doxycycline. §Higher IgG concentration after administration of intravenous immunoglobulin. ¶Bactec FX Blood Culture System (Becton, Dickinson and Company, https://www.bd.com).

**Figure 1:**
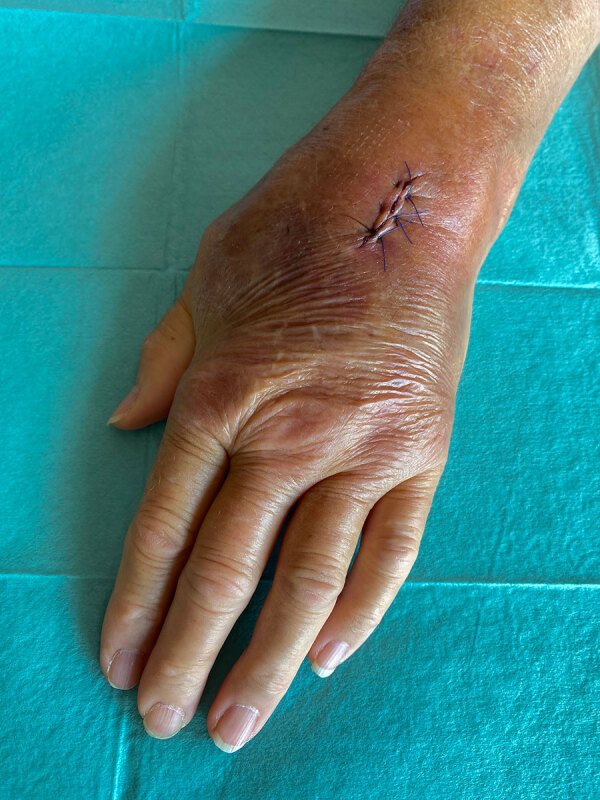
Surgical site after treatment for *Mycoplasma arginini* cellulitis, tenosynovitis, and arthritis in kidney transplant recipient, Slovenia, 2024. We observed swelling, redness, and restricted joint mobility in the left wrist before surgery.

**Figure 2:**
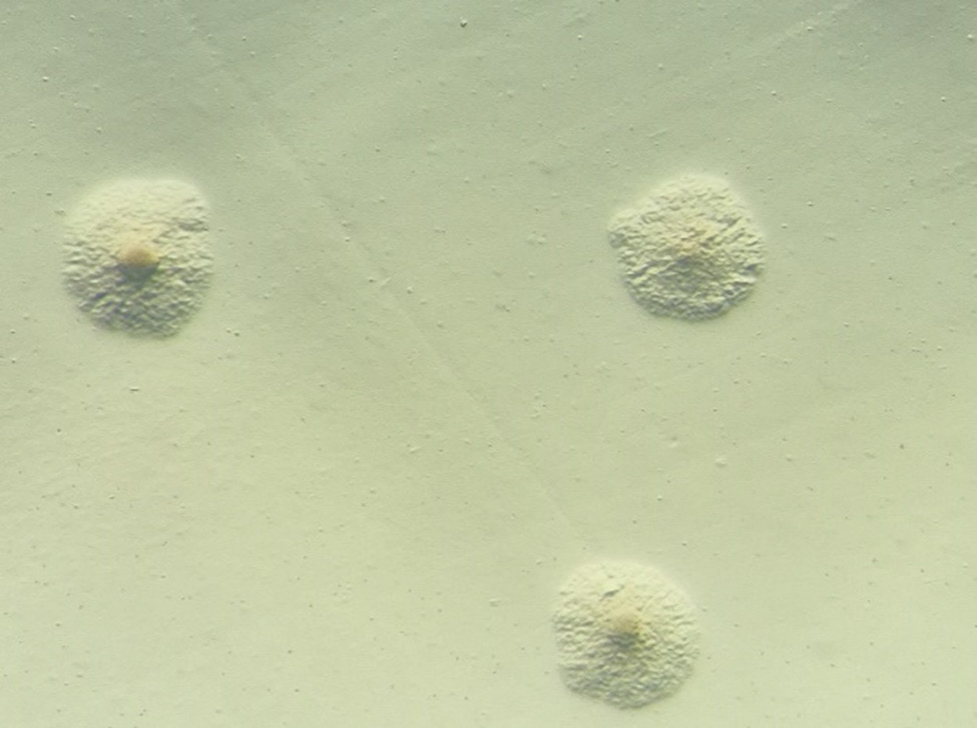
Stereomicroscopy of *Mycoplasma arginini* culture from the patient’s synovial fluid sample, showing small colonies with a characteristic fried egg appearance, with dense central core surrounded by a lighter peripheral zone. Original magnification ×40.

At admission, doctors initiated intravenous flucloxacillin therapy. The next day, doctors added doxycycline after an atypical infection was suspected. Surgical intervention on the radiocarpal joint included irrigation and the collection of synovial fluid and a biopsy specimen for microbiological analysis (Figure 1). Broad-range bacterial PCR with sequencing from synovial fluid identified *M. arginini* with 99.5% sequence identity; therefore, the sample was plated on arginine-enriched A8 agar. After 4 days, stereomicroscopy showed small colonies with a characteristic fried egg appearance (Figure 2).

After identification of *Mycoplasma* spp. from synovial fluid, we discontinued flucloxacillin and added moxifloxacin. The combination of doxycycline and moxifloxacin led to clinical improvement that allowed de-escalation to doxycycline monotherapy after 8 days. We treated severe hypogammaglobulinemia (IgG 2.7 g/L) with intravenous immunoglobulin on day 10 after admission. We discontinued mycophenolate on day 6 after confirmation of *M. arginini* infection. Ravulizumab, last administered 6 weeks before hospitalization, was postponed because of ongoing infection. Redness, swelling, and pain in the wrist and elbow resolved, and we discharged the patient on a 10-week course of doxycycline with outpatient follow-up. 

**Figure 3:**
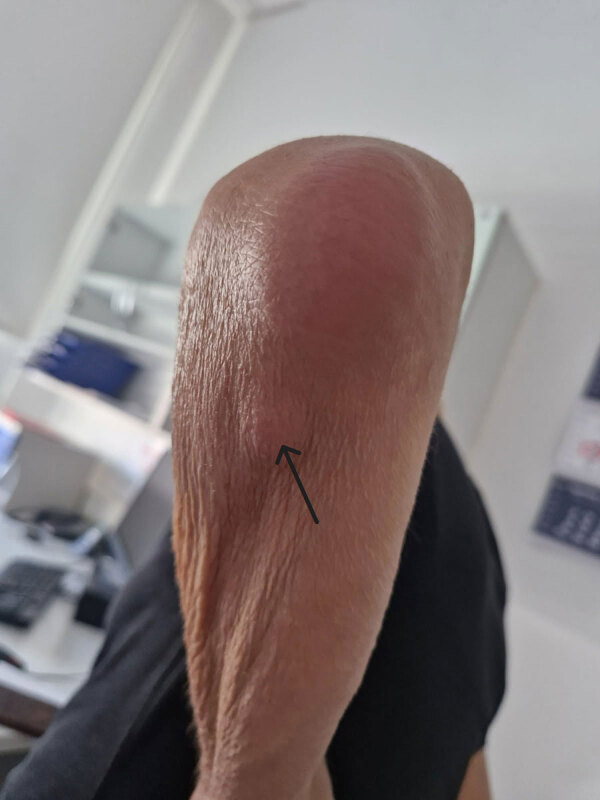
Subcutaneous nodular lesion (arrow) measuring 3 cm × 2.5 cm below elbow of patient in a case of *Mycoplasma arginini* cellulitis, tenosynovitis, and arthritis in kidney transplant recipient, Slovenia, 2024. The patient reported no pain in this nodule, and it was excised after discharge and 3 weeks of doxycycline treatment. Broad-range PCR confirmed *M. arginini* with 100% sequence identity, but *Mycoplasma* spp. culture was negative. Histopathology showed mild inflammatory changes consistent with bacterial infection.

After discharge and 3 weeks of doxycycline treatment, we excised a painless subcutaneous nodular lesion below the left elbow (Figure 3). Broad-range PCR performed on a sample from lesion confirmed *M. arginini* with 100% sequence identity. *Mycoplasma* spp. culture was negative. Histopathology showed mild inflammatory changes consistent with bacterial infection. Four months after ending the 10-week course of doxycycline treatment, the patient was doing well, without any local or systemic signs of *M. arginini* infection.

We tested oropharyngeal swab specimens from the patient’s 3 cats and 1 dog using a genus-specific mycoplasma PCR (*5*) and confirmed colonization with *M. gateae* and *M. maculosum*. We found that 1 cat had a mixed *Mycoplasma* infection, but we could not definitively confirm or rule out *M. arginini* in that cat. We gave a 2-week doxycycline course to the pets to treat *Mycoplasma*, although reinfection remained possible because they moved indoors and outdoors.

Only a few documented published cases support zoonotic potential of *M. arginini*, particularly in immunocompromised patients. Those cases include reports of disseminated infection in a slaughterhouse worker with advanced non-Hodgkin lymphoma (*2*), septicemia with polyarthritis in another therapy-resistant non-Hodgkin lymphoma patient who had close contact with several cats (*3*), a disseminated infection in a young bodybuilder with a history of use of nutritional supplements derived from animal materials of uncontrolled origin (*6*), and a deep tissue infection in a hunter with an open femur fracture from a lion attack (*7*). Common among those cases were profound immunosuppression and consumption of animal products or close contact with animals, as seen in our patient.

Research indicates that *M. arginini* poses little risk to persons with healthy immune systems. A study of 22 persons at occupational risk for *Mycoplasma* infections (e.g., veterinarians, farmers, and slaughterhouse workers) found no substantial infection risk in those with normal immune function, suggesting that human exposure to *M. arginini* is generally not a clinical concern for immunocompetent persons (*8*). Most documented cases, including the patient we report, involve immunocompromised persons, often with hypogammaglobulinemia, which is a known risk factor for infections caused by *Mycoplasma* spp. (*4*).

The optimal treatment for *M. arginini* infection remains uncertain because of its rarity in humans. Reported cases are typically managed with long-term courses of antimycoplasmal antibiotic drugs, such as macrolides, quinolones, and tetracyclines (*3*,*6*,*7*). In 1 instance, a patient treated with erythromycin ultimately died from the infection, and postmortem testing showed erythromycin-resistant *M. arginini* (*2*). That testing suggests that broader-spectrum antibiotic drugs may be more effective as initial treatment, particularly because erythromycin resistance is well documented in several *Mycoplasma* species (*9*,*10*).

Because *Mycoplasma* bacteria lack a cell wall, infections are best addressed with intracellular-acting antibiotic drugs such as tetracyclines or macrolides. Dual antibiotic drug therapy has been applied in specific cases to improve treatment outcomes, especially in immunocompromised persons or patients with disseminated infections (*11*). Treatment duration remains unclear. A meta-analysis of septic arthritis caused by *Mycoplasma* spp. reported therapy durations ranging from 2 weeks to >2 years. Clinical improvement, normalization of inflammatory markers, and negative reculture results should guide discontinuation (*11*).

The case we report also raises questions about persistently elevated β-d-glucan levels, which decreased but did not normalize after treatment. Although *Mycoplasma* species are not commonly associated with β-d-glucan production, studies suggest some species, such as *M. agalactiae*, might produce it (*12*). The zoonotic aspect is also notable, considering the well-documented colonization of domestic animals by *M. arginini* and the patient’s history of cat bites, likely making her pets the source of infection.

## Conclusions

This case underscores the limitations of conventional microbiology for detecting fastidious pathogens and highlights the value of molecular diagnostics for ensuring rapid and accurate diagnoses and effective treatment. Early recognition and appropriate therapy are crucial for improving outcomes in rare but serious *Mycoplasma* infections. Clinicians should consider *M. arginini* in the differential diagnosis of indolent infections in immunocompromised patients, particularly those with hypogammaglobulinemia and close animal contact.

Appendix Additional information for *Mycoplasma arginini* cellulitis, tenosynovitis, and arthritis in a kidney transplant recipient, Slovenia, 2024.

## References

[1] Chalker VJ. Canine mycoplasmas. Res Vet Sci. 2005;79:1–8. 10.1016/j.rvsc.2004.10.002 15894017

[2] Yechouron A, Lefebvre J, Robson HG, Rose DL, Tully JG. Fatal septicemia due to *Mycoplasma arginini*: a new human zoonosis. Clin Infect Dis. 1992;15:434–8. 10.1093/clind/15.3.434 1520790

[3] Watanabe M, Hitomi S, Goto M, Hasegawa Y. Bloodstream infection due to *Mycoplasma arginini* in an immunocompromised patient. J Clin Microbiol. 2012;50:3133–5. 10.1128/JCM.00736-12 22785195 PMC3421792

[4] Roifman CM, Rao CP, Lederman HM, Lavi S, Quinn P, Gelfand EW. Increased susceptibility to *Mycoplasma* infection in patients with hypogammaglobulinemia. Am J Med. 1986;80:590–4. 10.1016/0002-9343(86)90812-0 3963038

[5] Lierz M, Hagen N, Harcourt-Brown N, Hernandez-Divers SJ, Lüschow D, Hafez HM. Prevalence of mycoplasmas in eggs from birds of prey using culture and a genus-specific mycoplasma polymerase chain reaction. Avian Pathol. 2007;36:145–50. 10.1080/03079450701213347 17479375

[6] Silló P, Pintér D, Ostorházi E, Mazán M, Wikonkál N, Pónyai K, et al. Eosinophilic Fasciitis associated with *Mycoplasma arginini* infection. J Clin Microbiol. 2012;50:1113–7. 10.1128/JCM.05568-11 22189109 PMC3295179

[7] Prayson MJ, Venkatarayappa I, Srivastava M, Northern I, Burdette SD. Deep infection with *Mycoplasma arginini* in an open femur fracture secondary to an African lion bite: a case report. Inj Extra. 2008;39:243–6. 10.1016/j.injury.2007.12.009

[8] Sillis M. *Mycoplasma arginini*—a new human zoonosis? Clin Infect Dis. 1994;18:488–488. 10.1093/clinids/18.3.488 8011852

[9] Hannan PCT. Guidelines and recommendations for antimicrobial minimum inhibitory concentration (MIC) testing against veterinary mycoplasma species. International Research Programme on Comparative Mycoplasmology. Vet Res. 2000;31:373–95. 10.1051/vetres:2000100 10958240

[10] Okazaki N, Narita M, Yamada S, Izumikawa K, Umetsu M, Kenri T, et al. Characteristics of macrolide-resistant *Mycoplasma pneumoniae* strains isolated from patients and induced with erythromycin in vitro. Microbiol Immunol. 2001;45:617–20. 10.1111/j.1348-0421.2001.tb01293.x 11592636

[11] Chen Y, Huang Z, Fang X, Li W, Yang B, Zhang W. Diagnosis and treatment of mycoplasmal septic arthritis: a systematic review. Int Orthop. 2020;44:199–213. 10.1007/s00264-019-04451-6 31792575

[12] Gaurivaud P, Baranowski E, Pau-Roblot C, Sagné E, Citti C, Tardy F. *Mycoplasma agalactiae* secretion of β-(1→6)-glucan, a rare polysaccharide in prokaryotes, is governed by high-frequency phase variation. Appl Environ Microbiol. 2016;82:3370–83. 10.1128/AEM.00274-16 27037120 PMC4959233

