# Supplementary material for: Mycoplasma arginini Cellulitis, Tenosynovitis, and Arthritis in Kidney Transplant Recipient, Slovenia, 2024
Source: Emerg Infect Dis. 2025 Jun;31(6):1207–10. doi: 10.3201/eid3106.250149 (PMC12123927; doi:10.3201/eid3106.250149)
Supplement: Appendix — Additional information for Mycoplasma arginini cellulitis, tenosynovitis, and arthritis in a kidney transplant recipient, Slovenia, 2024. [file 25-0149-Techapp-s1.pdf]

Article DOI: https://doi.org/10.3201/eid3106.250149

EID cannot ensure accessibility for supplementary materials supplied by authors. Readers who have difficulty accessing supplementary content should contact the authors for assistance.

Mycoplasma arginini Cellulitis, Tenosynovitis, and Arthritis in Kidney Transplant Recipient, Slovenia, 2024

Appendix

Supplemental Materials

Identification, Isolation, and Whole-Genome Sequencing

Broad-range PCR targeting the V3/V4 region of the 16S rRNA gene (Molzym, https://www.molzym.com/) from synovial fluid identified *M. arginini* with 99.5% sequence identity confirmed through sequence analysis by using the CLC Main Workbench (QIAGEN) and National Center for Biotechnology Information BLAST (https://www.ncbi.nlm.nih.gov).

After successful molecular identification with broad-range PCR, *Mycoplasma* culture was conducted by inoculating 50 µL of synovial fluid onto an arginine-enriched A8 agar plate, which was incubated under anaerobic conditions at 35°C. After 4 days, small colonies with a characteristic “fried egg” appearance were observed with stereomicroscopy. These colonies were subcultured by using the Mycofast Screening Revolution assay (Elitech, France), based on liquid broth cultures as per the manufacturer's instructions. The assay was negative for urea hydrolysis but positive for arginine utilization after 48 hours of incubation. Additional testing with the Allplex STI Essential PCR Assay (Seegene, Korea) ruled out *Mycoplasma hominis* and other urogenital pathogens. Furthermore, PCR analysis with the Universal Mycoplasma Detection Kit (30–1012K; ATCC, USA) confirmed the presence of *Mycoplasma* DNA. This kit is able to detect a wide range of Mollicute, including *M. arginini*. The presence of *M. arginini* was verified by PCR for Mollicute based on the use of a 16S rRNA general primer pair (GPO3F and MGSO), followed by sequence analysis.

Additionally, routine synovial fluid culture was positive after 1 week of anaerobic incubation at 35°C, when bacterial growth was observed on Schaedler agar (bioMérieux, https://www.biomerieux.com). Colonies were identified as *M. arginini* with an identification score of 1.81 by using routine matrix-assisted laser desorption ionization-time of flight mass spectrometry (Bruker Daltonics, https://www.bruker.com). Tissue biopsy culture remained negative.

A pure *Mycoplasma* culture from A8 agar plates was used for DNA extraction with DNeasy Blood and Tissue kit (Qiagen, Germany) according to manufacturer's instructions.

The DNA library was prepared with Illumina DNA Prep according to the manufacturer’s instructions and sequenced with NextSeq550 (Illumina) by using paired-end chemistry with the read length of 2 × 149 bp. Obtained short reads were quality filtered by using fastp, removing low-quality reads and adaptor sequences (*1*). Reads were classified based on k-mer content with KrakenUniq (*2*), against the NCBI Nucleotide database collection. Genome assembly into contigs was performed with SPAdes v3.15.5 (*3*). Pairwise comparison of average nucleotide identity of the assembly and 17 other publicly available *M. arginini* genomes was performed by using FastANI (*4*). All 18 genomes were additionally uploaded to the Type Strain Genome Server (TYGS) for further whole genome–based taxonomic analysis (*5*).

The assembled genome consisted of 16 contigs above the size of 500 bp, with the total size of 637,600 bp, GC content of 26.1%, and a mean depth of 670×. Genome annotation revealed 551 coding sequences and 35 noncoding RNA genes.

Read classification showed that 80% of the reads were classified as *M. arginini*, whereas 17% could not be classified, most likely because of belonging to a novel species/strain.

FastANI analysis showed an average pairwise identity of 94.15% (93.92%–94.40%) compared with other *M. arginini* strains, which is below the intraspecies threshold of 95% proposed by Jain et al. (*4*), whereas the other strains' pairwise identities ranged from 97.99%–99.99%. Further taxonomic analysis through TYGS supported these data as well by designating our isolate to a different species and subspecies cluster than the rest of the *M. arginini* strains (Appendix Figure 2).

Analysis of lymphocyte populations in peripheral blood

Analysis of lymphocyte populations (T, B, Th, Tc, NK):

(Normal values apply to the patient's age.)

Percentage of T lymphocytes (CD3) among lymphocytes: 96%

Concentration of T lymphocytes (CD3) among lymphocytes: 1.662 × 10^9 cells/L; normal value (reference range: 0.7–1.9 × 10^9 cells/L)

Percentage of B lymphocytes (CD19): 0%

Concentration of B lymphocytes (CD19): 0.002 × 10^9 cells/L; decreased value (reference range: 0.1–0.4 × 10^9 cells/L)

Percentage of helper T cells (CD4) among T lymphocytes (CD3): 33%

Concentration of helper T cells (CD4) among T lymphocytes (CD3): 0.567 × 10^9 cells/L; normal value (reference range: 0.4–1.3 × 10^9 cells/L)

Percentage of cytotoxic T cells (CD8) among T lymphocytes (CD3): 61%

Concentration of cytotoxic T cells (CD8) among T lymphocytes (CD3): 1.053 × 10^9 cells/L; increased value (reference range: 0.2–0.7 × 10^9 cells/L)

Ratio of helper T cells to cytotoxic T cells (CD4/CD8): 0.54

Percentage of activated T lymphocytes (HLA-DR): 55%

Concentration of activated T lymphocytes (HLA-DR): 0.914 × 10^9 cells/L; increased value (reference range: 0.04–0.2 × 10^9 cells/L)

Percentage of natural killer (NK) cells (CD16+ CD56): 3%

Concentration of natural killer (NK) cells (CD16+ CD56): 0.056 × 10^9 cells/L; decreased value (reference range: 0.1–0.4 × 10^9 cells/L)

T lymphocytes: differentiation:

Percentage of naïve (CD45RA) cells among T lymphocytes (CD4): 30%

Concentration of naïve (CD45RA) T lymphocytes (CD4): 0.170 × 10^9 cells/L; decreased value (reference range: 0.23–0.77 × 10^9 cells/L)

Percentage of memory (CD45RA−) cells among T lymphocytes (CD4): 70%

Concentration of memory (CD45RA−) T lymphocytes (CD4): 0.397 × 10^9 cells/L; normal value (reference range: 0.24–0.70 × 10^9 cells/L)

Percentage of T lymphocytes CD4 RTE (CD45RA+ CD31+): 14%

Concentration of T lymphocytes CD4 RTE (CD45RA+ CD31+): 0.079 × 10^9 cells/L; normal value (reference range: 0.042–0.399 × 10^9 cells/L)

Percentage of TCR α/β DNT (CD4− CD8−) cells among T lymphocytes (CD3): 4%; normal value (reference range: up to 4%)

Percentage of TCR γ/δ DNT (CD4− CD8−) cells among T lymphocytes (CD3): 7%; increased value (reference range: up to 5%)

Percentage of TCR α/β cells among T lymphocytes (CD3): 92%

Percentage of TCR γ/δ cells among T lymphocytes (CD3): 8%

Percentage of helper T cells expressing cIFN-γ (cIFN-γ/+ CD4+): 31.6%; increased value (reference range: 10%–26%)

Percentage of helper T cells expressing cIL-4 (cIL-4+ CD4+): 3.1%; normal value (reference range: 2%–5%)

Percentage of helper T cells expressing cIL-17 (cIL-17+ CD4+): 1.5%; normal value (reference range: 1%–5%)

Percentage of helper T cells expressing CD25 (CD25++ CD4+): 1.4%; normal value (reference range: 1%–5%)

Percentage of activated helper T cells (CD25+ CD4+): 17.1%; normal value (reference range: 2.4%–35%)

B lymphocytes: differentiation

Unregulated B lymphocytes (CD21low CD38low CD19+): percentage of B lymphocytes: 27.4%; increased value (reference range: 0.4%–10.3%)

Mature B lymphocytes (CD21+ CD19+): percentage of B lymphocytes: 48.1%; decreased value (reference range: 91.1%–100%)

Memory B lymphocytes (CD27+ CD19+): percentage of B lymphocytes: 82.9%; increased value (reference range: 7.6%–40.7%)

Nonswitched memory B lymphocytes (CD27+ IgM+ IgD+ CD19+): percentage of B lymphocytes: 19.8%; increased value (reference range: 0%–8.5%)

IgM-only positive memory B lymphocytes (CD27+ IgM+ IgD– CD19+): percentage of B lymphocytes: 9.8%; increased value (reference range: 0%–5.3%)

Class-switched memory B lymphocytes (CD27+ IgM– IgD– CD19+): percentage of B lymphocytes: 63.1%; increased value (reference range: 2.3%–26.5%)

IgM+ cells among B lymphocytes (IgM+ CD19+): percentage of B lymphocytes: 30.6%; increased value (reference range: 5.4%–20.9%)

Transitional (preplasmacyte) B lymphocytes (CD38+ IgM+ CD19+): percentage of B lymphocytes: 0.9%; decreased value (reference range: 1.2%–50.7%)

Plasmablasts (CD38+ IgM– CD19+): percentage of B lymphocytes: 13.2%; normal value (reference range: 4.1%–42.2%)

QuaniFERON Monitor test

The cell-mediated immune response was assessed by using the QuantiFERON Monitor test, which yielded a result of 270 IU/mL, indicating a moderate level of cell-mediated immunity.

Reference values:

<15 IU/mL: weak cellular immunity (low IFN-γ-response after stimulation with activators of innate and adaptive immunity)

15–1,000 IU/mL: moderate cellular immunity (moderate IFN-γ-response after stimulation with activators of innate and adaptive immunity)

>1,000 IU/mL: strong cellular immunity (strong IFN-γ-response after stimulation with activators of innate and adaptive immunity)

References

1. Chen S. Ultrafast one-pass FASTQ data preprocessing, quality control, and deduplication using fastp. iMeta. 2023;2:e107. PubMed https://doi.org/10.1002/imt2.107

2. Breitwieser FP, Baker DN, Salzberg SL. KrakenUniq: confident and fast metagenomics classification using unique k-mer counts. Genome Biol. 2018;19:198. PubMed https://doi.org/10.1186/s13059-018-1568-0

3. Prjibelski A, Antipov D, Meleshko D, Lapidus A, Korobeynikov A. Using SPAdes de novo assembler. Curr Protoc Bioinformatics. 2020;70:e102. PubMed https://doi.org/10.1002/cpbi.102

4. Jain C, Rodriguez-R LM, Phillippy AM, Konstantinidis KT, Aluru S. High throughput ANI analysis of 90K prokaryotic genomes reveals clear species boundaries. Nat Commun. 2018;9:5114. PubMed https://doi.org/10.1038/s41467-018-07641-9

5. Meier-Kolthoff JP, Göker M. TYGS is an automated high-throughput platform for state-of-the-art genome-based taxonomy. Nat Commun. 2019;10:2182. PubMed https://doi.org/10.1038/s41467-019-10210-3

6. Letunic I, Bork P. Interactive Tree of Life (iTOL) v6: recent updates to the phylogenetic tree display and annotation tool. Nucleic Acids Res. 2024;52:W78–82. PubMed https://doi.org/10.1093/nar/gkae268

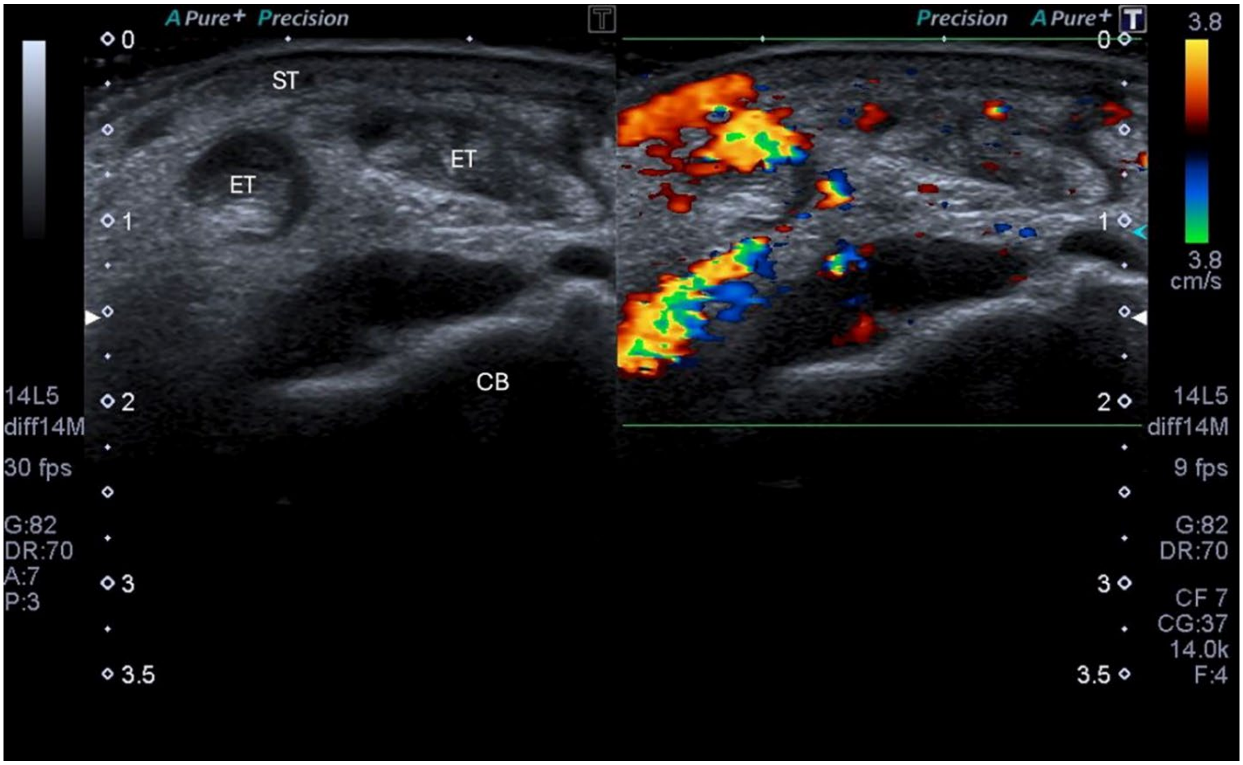

Appendix Figure 1. Ultrasound of the soft tissues of the left forearm revealed thickened and hyperemic subcutaneous tissue (ST) with densified fat containing fluid strands, consistent with ultrasound signs of cellulitis. A fluid layer was observed near the carpal bones (CB) and radiocarpal joint, indicative of arthritis. Additionally, along the extensor tendons (ET) of the fingers on the dorsum of the hand, a fluid layer was present, suggesting tenosynovitis.

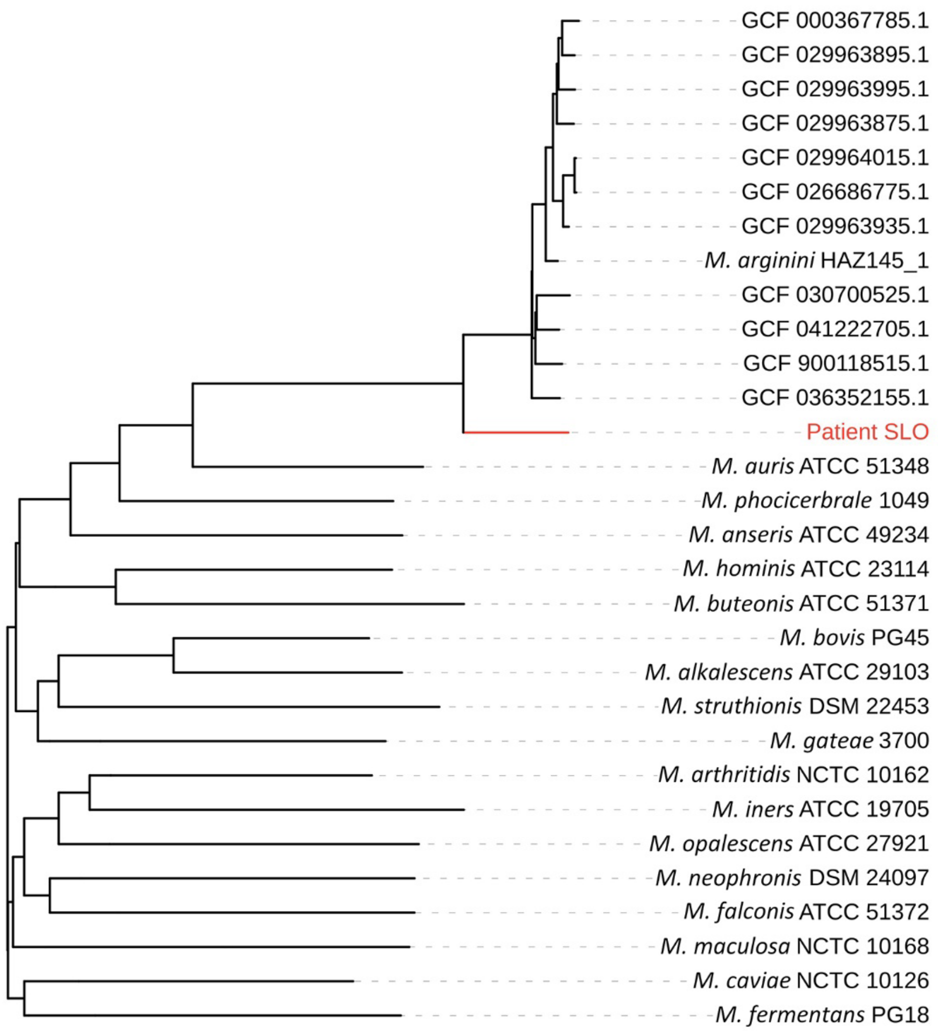

Appendix Figure 2. Type Strain Genome Server result for the M. arginini genome set. Tree inferred with FastME 2.1.6.1 from GBDP (Genome BLAST Distance Phylogeny) distances calculated from sequences and visualized with iTOL (*6*).
